# Clonal origin of *KMT2A* wild-type lineage-switch leukemia following CAR-T cell and blinatumomab therapy

**DOI:** 10.1038/s43018-023-00604-0

**Published:** 2023-07-20

**Authors:** Tim H. H. Coorens, Grace Collord, Taryn D. Treger, Stuart Adams, Emily Mitchell, Barbara Newman, Gad Getz, Anna L. Godfrey, Jack Bartram, Sam Behjati

**Affiliations:** 1 Broad Institute of MIT and Harvard, Cambridge, MA USA; 2 Great Ormond Street Hospital for Children, London, UK; 3 Wellcome Sanger Institute, Hinxton, UK; 4 Cambridge University Hospitals NHS Foundation Trust, Cambridge, UK; 5 Department of Paediatrics, University of Cambridge, Cambridge, UK; 6 Cancer Center and Department of Pathology, Massachusetts General Hospital, Boston, MA USA; 7 Harvard Medical School, Boston, MA USA

**Keywords:** Leukaemia, Cancer genomics, Paediatric cancer, Cancer

## Abstract

Children with acute lymphoblastic leukemia (ALL) undergoing anti-CD19 therapy occasionally develop acute myeloid leukemia (AML). The clonal origin of such lineage-switch leukemias^1–4^ remains unresolved. Here, we reconstructed the phylogeny of multiple leukemias in a girl who, following multiply relapsed ALL, received anti-CD19 cellular and antibody treatment and subsequently developed AML. Whole genome sequencing unambiguously revealed the AML derived from the initial ALL, with distinct driver mutations that were detectable before emergence. Extensive prior diversification and subsequent clonal selection underpins this fatal lineage switch. Genomic monitoring of primary leukemias and recurrences may predict therapy resistance, especially regarding anti-CD19 treatment.

## Abstract (web summary)

Coorens et. al. perform whole-genome sequencing and phylogenetic analyses in a child patient with leukemia that underwent lineage switch following therapy, finding that the AML arose from a pre-existing clone, rather than the most recent ALL relapse.

## Main

**Fig. 1:**
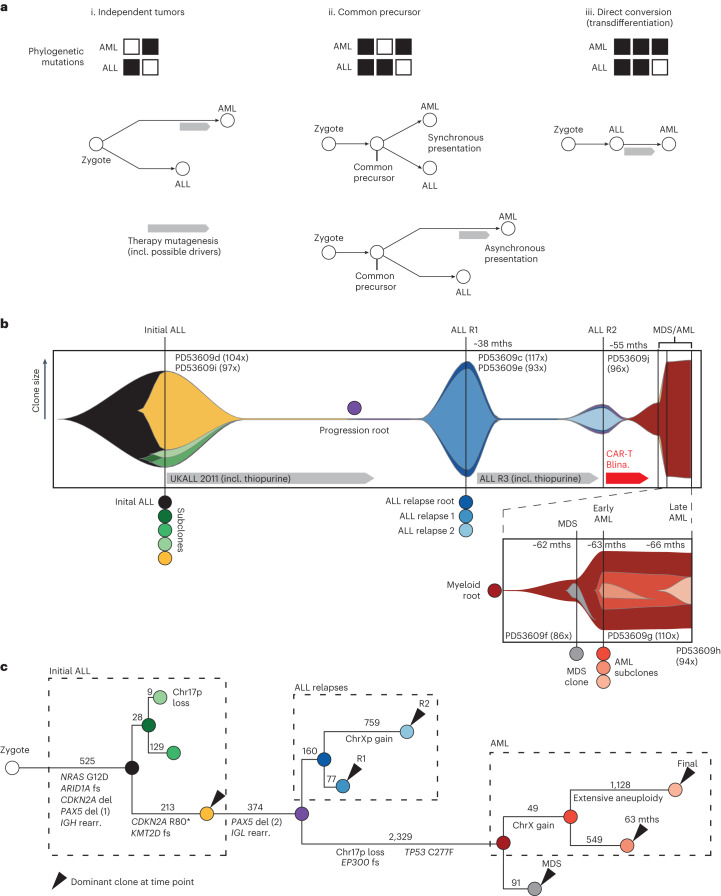
The clonal relationship between AML and ALL. **a**, Schematic overview of different relations between an ALL and subsequent AML from the same patient and corresponding mutational readouts. (i) The AML and ALL may be clonally unrelated malignancies, sharing no mutations, as is the case for treatment-related AML (t-AML). (ii) They may arise from a common precursor cell, typified by shared somatic mutations likely including a first driver mutation, but is itself not a cancer cell. (iii) The AML may derive from the ALL, which has undergone a true lineage switch through transdifferentiation, typified by the AML harboring all somatic mutations of the primary ALL. Note that asynchronous presentations of ALL and AML may involve mutations in the second lesion induced by the therapy for the first lesion. **b**, A fish plot showing the clone size of the different ALL and AML clones over the course of the clinical history of the patient. Number in parentheses after sample identifier refers to the genome-wide coverage of sequencing. mths, months; MDS, myelodysplastic syndrome. **c**, Reconstructed phylogeny of clones with annotated putative driver mutations and CNVs. Number of single base substitution mutations underlying each branch of the phylogenetic tree are denoted above the branch. Dashed lines group clones into initial ALL, ALL relapses and AML relapses. fs, frameshift; del, deletion; rearr., rearrangement.

Children with ALL occasionally develop a phenotypically distinct AML, which may be a clonally independent, second malignancy or represent a phenotypic transdifferentiation (‘class’ or ‘lineage’ switch) (Fig. 1a). Lineage switch is a rare phenomenon. It is more prevalent in leukemias underpinned by drivers that confer phenotypic plasticity, particular rearrangements of the *KMT2A* (*MLL*) gene^1–3^. Furthermore, lineage switch seems to occur more commonly in children with ALL treated with chimeric antigen receptor T (CAR-T) cells or antibody therapy (such as blinatumomab) targeting the principal lineage marker of B-cell lymphoblasts, CD19. In this context, lineage switch refers to a transdifferentiation from a lymphoid to a myeloid phenotype, rather than the emergence of CD19^−^ ALL expressing alternative CD19 transcripts^4^. In the era of precision therapies against CD19, understanding risk factors for lineage switch is of particular importance, as it may identify individuals who are more suitable for cytotoxic chemotherapy, including allogeneic stem cell transplantation, rather than blinatumomab or CAR-T treatment.

To understand the pathogenesis of any lineage-switch leukemia, it is essential to unambiguously determine the phylogenetic relationship with the antecedent leukemia. Several possibilities are illustrated below (Fig. 1a). The leukemias may represent completely independent malignancies, possibly due to germline predisposition. Alternatively, it is conceivable that both leukemias arise from a common precursor yet become clinically apparent at different time points. Also, lineage-switch leukemia may represent a direct conversion of a lymphoblast to myeloblast. Further important questions that we sought to answer include the timing of leukemia development (simultaneous mixed phenotype acute leukemia^5^ versus asynchronous^6^) and whether ALL treatment may have directly caused the subsequent myeloid leukemia through chemotherapy-induced mutagenesis. In any of these scenarios, the phylogenetic relationship between leukemias can be resolved through somatic mutations, which serve as a barcode of clonal development; however, it is critical to obtain a complete readout of somatic variants through whole-genome sequencing (WGS), as partial readouts may lead to the erroneous resolution of clonal relationships.

In clinical practice, lineage-switch leukemia is conventionally diagnosed based on limited genetic evidence, such as V(D)J rearrangements or specific cytogenetic changes that are insufficient to establish clonal phylogenies. Similarly, previous studies of phenotypically divergent leukemias have not captured genome-wide mutations across serial disease time points^7,8^, thus precluding precise resolution of the clonal relationship between malignancies. Here, we studied the phylogeny of a lineage-switch leukemia that occurred in a child following anti-CD19 cellular and antibody therapy, based on WGS to an average of 100× coverage from eight samples spanning six key stages of the disease.

The clinical history of the child is shown in Fig. 1b. In brief, a 3-year-old girl developed precursor-B-ALL with no unusual morphological or phenotypic features (as assessed by flow cytometry) and without common cytogenetic aberrations. Initially classified as National Cancer Institute standard risk, treatment was intensified at the end of induction due to raised measurable residual disease levels. Thereafter, the child achieved and maintained remission until ~3 years after the initial diagnosis. At this point, an isolated bone-marrow relapse occurred, which was phenotypically identical to the first leukemia. She was treated with a non-myeloablative strategy and achieved remission after induction. About 1.5 years into relapse treatment, she experienced a second relapse with phenotypically unchanged blasts, this time with combined central nervous system and bone-marrow disease. She achieved short-lived remissions following anti-CD19 CAR-T cell and then blinatumomab therapy. Seven months after her second B-ALL relapse, she developed AML, which was phenotypically distinct from the ALL and proved resistant to further treatment.

We performed WGS of eight samples (bone marrow or leukemic blood) from six different time points with an average coverage of approximately 100×, detailed in Fig. 1b. We called all classes of mutations (single-nucleotide variants (SNVs), short insertions and deletions (indels), copy-number variants (CNVs) and structural variants (SVs)), using an established variant calling pipeline, as described previously^9–11^. Our analysis focused on the timing and clonal origin of the AML lineage. Our analysis of the data revealed that the AML was a direct descendant of the primary ALL, supporting direct conversion of a lymphoid to myeloid cell (Fig. 1a).

**Extended Data Fig. 1:**
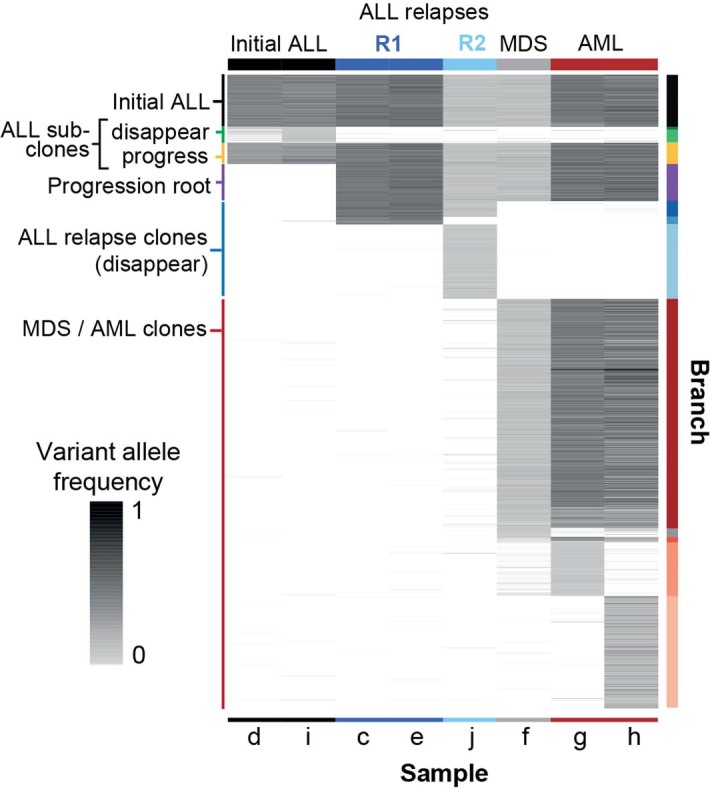
Heatmap of VAFs. Heatmap of the variant allele frequency of all identified substitutions across all samples. Branch colors correspond to Fig. 1b–c.

**Extended Data Fig. 2:**
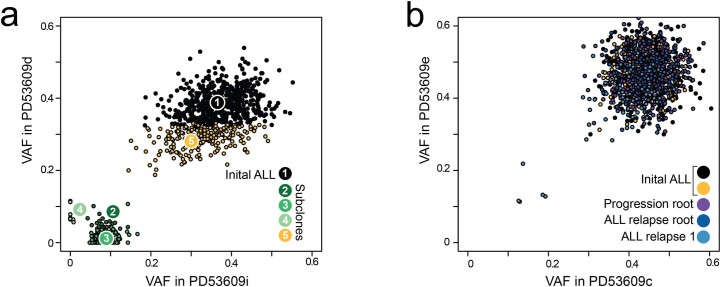
VAF plots in replicate samples. Plot of the variant allele frequency of mutations between different samples from the same time point: initial ALL (**a**) and All relapse 1 (**b**). Mutations are coloured by their branch assignment.

Analysis of WGS obtained from the initial (monophenotypic) ALL revealed a diverse clonal composition (Fig. 1c and Extended Data Figs. 1 and 2). The ancestral clone harbored four leukemogenic events: an activating hotspot mutation in *NRAS* (*G12D*) and hemizygous loss of three genes *CDKN2A*, *PAX5* and *ZCCHC7*. Of note, *PAX5* and *ZCCHC7* are adjacent genes and were truncated by the same 231-kb deletion. There were two further lineages: a smaller clone (accounting for 20% in the sample PD53609d), characterized by deletion of chromosome 17p (and thus of the *TP53* gene) and a larger clone (75% in PD53609d and 88% in PD53609i) defined by a truncating *KMT2D* mutation and inactivation of the remaining allele of *CDKN2A* (R80*). This larger clone gave rise to the progression lineage that generated subsequent leukemias, whereas the lineage with 17p loss was no longer detectable at later time points. All ALL clones shared the same VDJ rearrangements of the immunoglobulin heavy chain (*IGH*) and lacked further rearrangements of immunoglobulin λ (*IGL)* or κ (*IGK*) light chains.

**Fig. 2:**
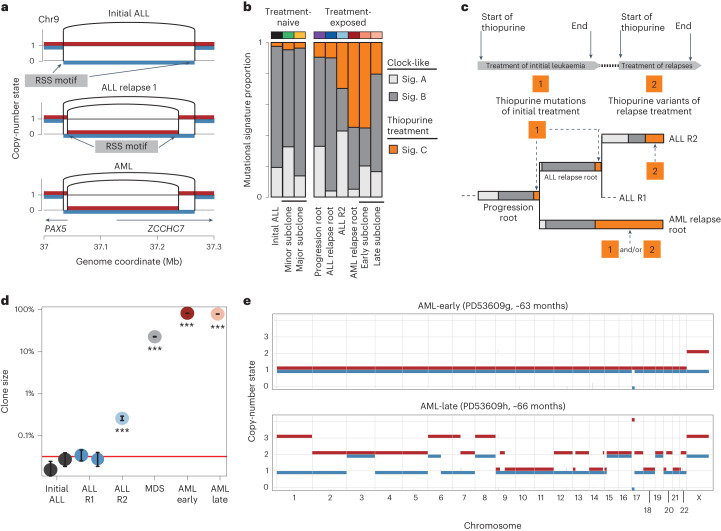
Mutational patterns across leukemia samples. **a**, Copy-number state in the *PAX5*-*ZCCHC7* locus, revealing a single loss in the initial ALL and a subsequent second hit in the relapse samples with nearby, but unique break points resulting in a constitutive loss of *PAX5* and *ZCCHC7*. Both deletions were flanked by recombination signal sequence (RSS) motifs, indicating that these deletions were likely RAG-mediated. **b**, Exposures of the three identified signatures in this patient, with signature A corresponding to COSMIC reference SBS1, signature B to SBS5 and signature C to SBS87. The latter is associated with thiopurine exposure. Only branches with 100 SNVs or more are displayed. **c**, Schematic of treatment courses of the patient, along with thiopurine-related mutagenesis. As the progression root and the ALL relapse root (its descendant) harbor thiopurine-induced mutations, the split between the ALL and AML lineages must have happened during the treatment for the initial ALL. **d**, Estimated clone size of ancestral AML lineage across samples, as estimated from interrogating all mutant sites in diploid regions (*n* = 2,318) in the AML root branch (Methods). The red line indicates the background error rate estimated from 32 unmatched blood samples. Error bars denote the 95% CI around maximum likelihood binomial estimate of clone size. ***P < 0.001, one-sided binomial test with null hypothesis that number of variant counts is drawn from background error distribution. The exact *P* value for the ALL R2 sample is 1.5 × 10^−151^ and 0 for all AML-related samples. **e**, Genome-wide copy-number profile of the two AML samples, obtained 3 months apart, showing that the later AML acquired extensive aneuploidy in a short time, likely facilitated by the biallelic loss of *TP53*.

The progression lineage acquired further driver mutations before bifurcating into ALL relapse and AML lineages, namely loss of the remaining *PAX5* and *ZCCHC7* alleles, again disrupted by a single deletion of a short chromosomal segment (196 kb). Flanking the 3′ break point of this second deletion, as well as of the first *PAX5*-*ZCCHC7* and *CDKN2A* deletion, were canonical sequence motifs targeted by recombination-activating gene (RAG) proteins, which mediate V(D)J recombination (Fig. 2a; Methods). In addition, the emergence of the second *PAX5*-*ZCCHC7* deletion phylogenetically coincided with VJ recombination of *IGL* (Fig. 1c). This indicates that off-target activity of RAG, which is often responsible for rearrangements in healthy^12^ and malignant^13^ B cells, may underpin these structural leukemogenic variants, especially as RAG-mediated recombination is known to affect highly expressed genes involved in B-cell development and differentiation, such as *PAX5* and *ZCCHC7* (ref. ^13^).

**Extended Data Fig. 3:**
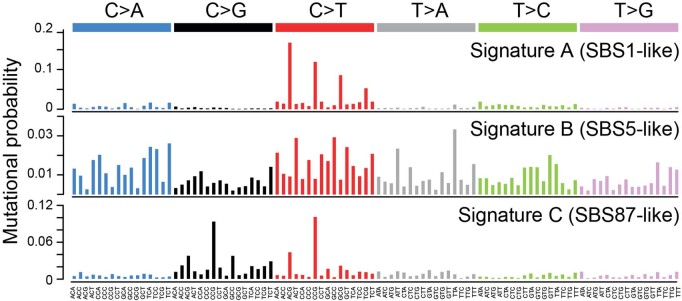
Mutational signatures. Profiles of the three identified mutational signatures in this patient, with Signature A corresponding to COSMIC reference SBS1, Signature B to SBS5 and Signature C to SBS87. The latter is associated with thiopurine exposure.

Following acquisition of these additional driver events, the relapse lineage bifurcated into separate ALL relapse and AML class-switch lineages. We were able to precisely time this bifurcation, based on the distribution of substitutions carrying the specific mutational imprint (signature C, corresponding to SBS87) of thiopurine exposure^9,14^, as defined by the trinucleotide context of base substitutions (Fig. 2b and Extended Data Fig. 3). The first ALL relapse clone carried private thiopurine variants, as well as thiopurine mutations shared with the AML lineage. This indicates that the relapse lineage diverged from the AML lineage at a time point after first thiopurine exposure (accounting for shared thiopurine mutations), but before the last dose of thiopurine (given the private thiopurine mutations) (Fig. 2c). Accordingly, the diversification must have arisen before the end of treatment of the original ALL. Of note, none of the variants attributable to thiopurine treatment generated apparent driver events.

Next, we examined the two ALL relapses, expecting that the second relapse directly descended from the first relapse. We found, however, that relapses represented independent lineages that originated in a common, bifurcating precursor. During the treatment of relapses with cytotoxic agents, the AML lineage continued to acquire somatic mutations, as evidenced by the accumulation of additional thiopurine-related variants. We detected evidence of this AML lineage for the first time at second ALL relapse (R2) at a variant allele frequency (VAF) of 0.0013 (95% binomial confidence interval (CI) 0.011–0.0014), through recalling mutations in the AML root lineage and leveraging the statistical power derived from considering multiple variant loci simultaneously (Fig. 2d). In the next sample of bone marrow obtained following CAR-T and blinatumomab treatment, which lacked phenotypic evidence of either a lymphoid or myeloid blast population, this lineage had expanded (~23%). The expanded clone, in essence representing MDS, carried additional driver events, truncation of *EP300* and homozygous hotspot mutation of *TP53*. Within 2 weeks, peripheral myeloblasts appeared, accompanied by a transformed bone marrow that now harbored overt AML. From here, the AML clone further diversified, as seen in a marrow sample examined 3 months later. Within this short timeframe, the AML went from exhibiting a largely quiescent copy-number profile to extensive aneuploidy (Fig. 2e). At all time points, the AML retained the precise V(D)J recombination in IGH found in the initial ALL, as well as the VJ recombination sequence found in IGL, further supporting the idea that these myeloid cells are directly derived from lymphoid cells.

Detailed genomic investigations of leukemia of the past decade have revealed unexpected genetic diversity in leukemia, with sweeps of subclonal diversification and selection of pre-existing or the generation of de novo clones underpinning disease progression^15,16^. Here, we built a detailed phylogeny of a lethal leukemia from presentation through relapses to lineage switch, following anti-CD19 directed therapy, leveraging deep WGS from serial time points. Beyond extensive genetic diversification, the unexpected finding in our data was the indirect phylogenetic relationship between clones at every disease stage, diagnosis, ALL relapses and AML. While one clone was dominant at any one disease stage, none of these dominant clones, not even the two ALL relapses, was a direct linear descendant of one another. Accordingly, although derived from the initial ALL, the AML lineage did not represent the direct conversion of the most recent ALL relapse clone. Instead, the AML lineage emerged from a pre-existing lineage that evolved in the context of a genetically diversified leukemia. This indicates that cancer cells belonging to alternative lineages were present elsewhere, persisting through treatment and further accruing somatic mutations. This hints at a large reservoir of genetically diverse clones, which may have resisted therapy through specific driver events. An important limitation of our study is that it is based on a single patient and may not be representative of anti-CD19 therapy associated lineage switch, highlighting the need for longitudinal tissue banking to enable a general understanding of lineage-switch leukemia.

The clinical implications of our findings are twofold. The first pertains to whether any somatic genetic feature of the initial leukemia might have predicted therapy resistance, relapse or lineage switch. What is notable in this child’s case is the sizable expansion of several clones at diagnosis that harbored bona fide driver events. These included *CDKN2A*, which has been associated with poor prognosis and chemotherapy resistance in ALL^17^ and loss of *PAX5*, which encodes the master transcription factor controlling B-cell identity^18^ and may have mediated plasticity and the transition to a myeloid phenotype. A truncal driver event in the myeloid lineage was biallelic loss of TP53, which has been associated with therapy-related myeloid neoplasms^19^ and likely further contributed to disease progression.

Second, our findings raise the question whether treatment precipitated progression and lineage switch or whether the myeloid leukemia should be considered a second, therapy-related neoplasm. In every leukemia that followed the initial diagnosis, we detected therapy-related mutations; however, the probability of individual driver events being caused by thiopurine exposure was low. In fact, key leukemogenic events in the ultimately fatal lineage pre-existed at diagnosis. Some of the most truncal driver variants were caused by RAG-mediated mutagenesis, which continued to be active in at least the early progression lineage (as evidenced by further IGL rearrangement). This suggests that blast-intrinsic mechanisms contributed at least in part to disease progression, which, in the context of a plasticity-promoting *PAX5* deletion, may have pushed the progression lineage toward transdifferentiation.

At every time point, the progression of leukemia primarily represented the selection of pre-existing clones, most poignantly seen in the presence of the ultimately fatal lineage at second ALL relapse (time point R2). We therefore view the extensive genetic diversification as the key feature of this child’s disease which, under treatment pressure, led to the emergence of a lethal clone with lineage switch. These considerations lead us to propose that genomic monitoring by WGS of the primary tumor and relapses could have predicted a high risk to relapse and lineage switch. As WGS of childhood cancers is beginning to enter routine clinical practice, as, for example, available in England through the National Health Service, we will develop a precise phylogenetic understanding of lineage-switch leukemia that may translate into anticipatory treatment strategies, especially in progressive childhood leukemia.

## Methods

### Samples and sequencing

All human material was obtained from patients enrolled in the ethically approved study, ‘Investigating how childhood tumors and congenital disease develop’ (UK NHS National Research Ethics Service reference 16/EE/0394). DNA was extracted from fresh-frozen tumor samples or blood samples. Short insert (500 bp) genomic libraries were constructed and 150-bp paired-end sequencing clusters were generated on the Illumina NovaSeq platform. We sequenced eight samples from six different time points, which constituted all samples available for this patient. Hence, no statistical methods were used to predetermine sample sizes. An overview of samples, including the average sequence coverage is shown in Supplementary Table 1. Of note, two samples each were taken from the initial ALL (PD53609d and PD53609i) and the first ALL relapse (PD53609c and PD53609e). No data were excluded from the analyses. The investigators were not blinded to allocation during experiments and outcome assessment. Further information on research design is available in the Nature Research Reporting Summary linked to this article.

### DNA sequence processing, mutation calling and filtering

DNA sequences were aligned to the GRCh38 reference genome by the Burrows–Wheeler algorithm (BWA-MEM)^20^.

SNVs and indels were called against the reference genome using CaVEMan^21^ and Pindel^22^, respectively. Beyond the standard post-processing filters of CaVEMan, we removed variants affected mapping artifacts associated with BWA-MEM by setting the median alignment score of reads supporting a mutation as greater than or equal to 140 (ASMD ≥ 140) and requiring that fewer than half of the reads were clipped (CLPM = 0). In addition, variants that were disproportionately supported by low-quality bases or located near indels were removed. Filtered SNV and indel calls are reported in Supplementary Tables 2 and 3, respectively.

Across all samples from this patient, we force-called the SNVs and indels that were called in any sample, using a cutoff for read mapping quality (30) and base quality (25). Germline variants were removed using a one-sided binomial exact test on the number of variant reads and depth present across largely diploid samples, as previously described^11,23^. Resulting *P* values were corrected for multiple testing with the Benjamini–Hochberg method^24^ and a cutoff was set at *q* < 10^−5^. Using a β-binomial model of a site-specific error rate as previously employed^8^, we distinguished true presence of SNVs from support due to noise.

CNVs were called using ASCAT^25^ and Battenberg^26^. SVs were called using BRASS^27^.

### Clustering of SNVs and phylogeny reconstruction

We used the *n*-dimensional DPClust algorithm^26,28^ to cluster SNVs, incorporating their copy-number state as well as the purity and ploidy of each sample. As nDPclust does not complete for all eight samples simultaneously, we constructed a core clustering by using a sample from each time point (PD53609d for the initial ALL, PD53609d for the first ALL relapse, PD53609j for the second ALL relapse and PD53609g for the AML). This clustering was further enriched by running nDPClust for each of the time points in depth (PD53609c, PD53609e and PD53609j for the ALL relapses and PD53609f, PD53609g and PD53609h for the MDS/AML). The subclonal makeup of the initial ALL was disentangled by running single-dimension DPClust on PD53609d and PD53609i individually.

Based on the contribution of each mutation cluster to each sample and the various results of the clustering across samples, these mutational clusters, which effectively form branches of a phylogeny, were then organized into a phylogenetic tree by considering the pigeonhole principle, the contribution of daughter clades cannot exceed the contribution of the parental branch. Cluster information, as well as the assignment of SNVs to branches, can be found in Supplementary Table 2. A heat map of SNV VAFs along with the cluster annotation can be found in Extended Data Fig. 1.

For multiple samples taken from the same time point (PD53609d and PD53609i for the initial ALL and PD53609c and PD53609e for the first ALL relapse), the mutation VAFs along with clonal assignment are plotted in Extended Data Fig. 2.

### Mutational signature analysis

SNVs per branch were converted to counts of trinucleotide changes. From these, signatures were extracted using the R package SigFit (https://github.com/kgori/sigfit)^29^, resulting in three signatures (signature A, signature B and signature C). Compared to COSMIC reference mutational signatures, these corresponded to, but were not identical to, SBS1, SBS5 and SBS87, respectively (Extended Data Fig. 3). Trinucleotide profiles per signature are in Supplementary Table 4 and mutational signature exposures per branch are in Supplementary Table 5.

### Immunoglobulin rearrangement calling

Rearrangements in the immunoglobulin gene loci (*IGH*, *IGL* and *IGK*) were called using MiXCR (https://github.com/milaboratory/mixcr)^30^.

### Identification of recombination signal sequences

RAG-mediated deletions were called using a motif search for RSSs close to deletion break points^13^. The RSS motifs with the 12-bp or 23-bp spacer in the middle, along with 50 bp up- and downstream of each of the break points around the *PAX5*-*ZCCHC7* locus were passed to the FIMO algorithm (https://meme-suite.org/meme/doc/fimo.html), which calculates a probability of the presence of a motif, assuming a zero-order background model. Break point-adjacent sequences with a *P* value < 0.05 were taken as evidence for off-target RAG-mediated deletions. The output from FIMO is in Supplementary Table 6.

### Estimating clone size of ancestral AML lineage across samples

To assess whether the lineage ancestral to the AML relapses was present at a very low fraction in preceding ALL samples, we interrogated all variant sites that define this AML root branch, excluding variants affected by the 17p loss observed in the AML samples. For the remaining variant sites (*n* = 2,318), the number of variant-supporting reads and total number of reads covering these sites were extracted from the bam files, with a minimum base quality of 25 and a minimum mapping quality score of 40. To assess the inherent error rate of these sites, we interrogated the same sites in 32 unmatched blood samples from a previous study^31^, yielding a mean error rate of 1.6 × 10^−4^. To assess whether the observed number of variant counts were in line with the error rate, we performed a one-sided binomial test for the observations in each sample. The counts and resulting exact *P* values can be found in Supplementary Table 7.

### Reporting summary

Further information on research design is available in the Nature Portfolio Reporting Summary linked to this article.

## Supplementary information

Reporting Summary

Supplementary Table 1 Supplementary Tables 1–7.

## Data Availability

Raw sequencing data have been deposited in the European Genome–phenome Archive under study ID EGAD00001009161. Processed variant calls are in Supplementary Tables 2 and 3. Output files of mutational signature analyses are in Supplementary Tables 4 and 5. Outputs from FIMO on RSS sequence motif enrichment are in Supplementary Table 6. Outputs from FIMO on RSS sequence motif enrichment are in Supplementary Table 7. Source data for all figures are provided in the supplementary tables. Data on reference mutational signatures are available from the COSMIC database (https://cancer.sanger.ac.uk/signatures/). All other data supporting the findings of this study are available from the corresponding author on reasonable request.

## Code availability

Code used and described in this paper for analysis and visualization can be found online at https://github.com/TimCoorens/ALL_AML_switch.
