# Supplementary material for: Clonal origin of KMT2A wild-type lineage-switch leukemia following CAR-T cell and blinatumomab therapy
Source: Nat Cancer. 2023 Jul 20;4(8):1095–101. doi: 10.1038/s43018-023-00604-0 (PMC10447231; doi:10.1038/s43018-023-00604-0)
Supplement: Supplementary file 1 — Reporting Summary [file 43018_2023_604_MOESM1_ESM.pdf]

nature portfolio

Corresponding author(s): T Coorens, S Behjati

Last updated by author(s): Apr 4, 2023

Reporting Summary

Nature Portfolio wishes to improve the reproducibility of the work that we publish. This form provides structure for consistency and transparency in reporting. For further information on Nature Portfolio policies, see our Editorial Policies and the Editorial Policy Checklist.

Statistics

For all statistical analyses, confirm that the following items are present in the figure legend, table legend, main text, or Methods section.

n/a	Confirmed	
☐	☒	The exact sample size (*n*) for each experimental group/condition, given as a discrete number and unit of measurement
☐	☒	A statement on whether measurements were taken from distinct samples or whether the same sample was measured repeatedly
☐	☒	The statistical test(s) used AND whether they are one- or two-sided<br>*Only common tests should be described solely by name; describe more complex techniques in the Methods section.*
☒	☐	A description of all covariates tested
☒	☐	A description of any assumptions or corrections, such as tests of normality and adjustment for multiple comparisons
☐	☒	A full description of the statistical parameters including central tendency (e.g. means) or other basic estimates (e.g. regression coefficient) AND variation (e.g. standard deviation) or associated estimates of uncertainty (e.g. confidence intervals)
☐	☒	For null hypothesis testing, the test statistic (e.g. *F*, *t*, *r*) with confidence intervals, effect sizes, degrees of freedom and *P* value noted<br>*Give P values as exact values whenever suitable.*
☒	☐	For Bayesian analysis, information on the choice of priors and Markov chain Monte Carlo settings
☒	☐	For hierarchical and complex designs, identification of the appropriate level for tests and full reporting of outcomes
☒	☐	Estimates of effect sizes (e.g. Cohen's *d*, Pearson's *r*), indicating how they were calculated

Our web collection on statistics for biologists contains articles on many of the points above.

Software and code

Policy information about availability of computer code

Data collection: No software was used for data colelction

Data analysis:
- SNV variant calling: CaVEMan (https://github.com/cancerit/CaVEMan) (v.1.14.0)
- Indel calling: Pindel (https://github.com/cancerit/cgpPindel) (v.3.9.0)
- CNV calling: ASCAT (https://github.com/cancerit/ascatNgs) (v.4.4.1) and Battenberg (https://github.com/cancerit/cgpBattenberg) (v.3.5.3)
- Immunoglobulin rearrangement calling: MiXCR (https://github.com/milaboratory/mixcr) (v3.0.13)
- Mutational signature analysis: SigFit (https://github.com/kgori/sigfit) (v2.0.0)
- Recombination signal sequence motif analysis: FIMO (https://meme-suite.org/meme/doc/fimo.html) (v.5.5.2)
- Clonal reconstruction: DPClust (https://github.com/Wedge-lab/dpclust) (v.2.2.6)

Code used and described in this paper for analysis and visualization can be found online at https://github.com/TimCoorens/ALL_AML_switch.

For manuscripts utilizing custom algorithms or software that are central to the research but not yet described in published literature, software must be made available to editors and reviewers. We strongly encourage code deposition in a community repository (e.g. GitHub). See the Nature Portfolio guidelines for submitting code & software for further information.

Data

Policy information about availability of data

All manuscripts must include a data availability statement. This statement should provide the following information, where applicable:

- Accession codes, unique identifiers, or web links for publicly available datasets
- A description of any restrictions on data availability
- For clinical datasets or third party data, please ensure that the statement adheres to our policy

Raw sequencing data have been deposited in the European Genome-phenome Archive (EGA) under study ID EGAD00001009161. Processed variant calls can be found in Supplementary Tables 2-3. Output files of mutational signature analyses can be found in Supplementary Tables 4-5. Output from FIMO on RSS sequence motif enrichment can be found in Supplementary Table 6. Output from FIMO on RSS sequence motif enrichment can be found in Supplementary Table 7. Source data for all figures is provided in the Supplementary tables. Data on reference mutational signatures is available from the COSMIC database (https://cancer.sanger.ac.uk/signatures/). All other data supporting the findings of this study are available from the corresponding author on reasonable request.

Human research participants

Policy information about studies involving human research participants and Sex and Gender in Research.

Reporting on sex and gender	Patient was of the female sex.
Population characteristics	In brief, a three-year-old girl developed pre-B ALL with no unusual morphological or phenotypic features (as assessed by flow cytometry) at diagnosis, and without common cytogenetic aberrations. Initially classified as NCI standard risk, treatment was intensified at the end of induction, due to raised minimal residual disease levels. Thereafter, the child achieved and maintained remission until 38 months after the initial diagnosis. At this point, an isolated bone marrow relapse occurred which was phenotypically identical to the first leukemia. The child was treated with a non-myeloablative strategy and achieved remission after induction. Seventeen months into relapse treatment, she experienced a second relapse with phenotypically unchanged blasts, this time with combined central nervous system and bone marrow disease. She achieved short-lived remissions following anti-CD19 CAR-T cell and then antibody therapy. Seven months after her second B-ALL relapse, she developed AML, which was phenotypically distinct from the ALL and proved resistant to further treatment.
Recruitment	Patient was recruited through a clinical setting.
Ethics oversight	All human material was obtained from patients enrolled in the ethically approved study, “Investigating how childhood tumors and congenital disease develop” (UK NHS National Research Ethics Service reference 16/EE/0394).

Note that full information on the approval of the study protocol must also be provided in the manuscript.

Field-specific reporting

Please select the one below that is the best fit for your research. If you are not sure, read the appropriate sections before making your selection.

☒ Life sciences ☐ Behavioural & social sciences ☐ Ecological, evolutionary & environmental sciences

For a reference copy of the document with all sections, see nature.com/documents/nr-reporting-summary-flat.pdf

Life sciences study design

All studies must disclose on these points even when the disclosure is negative.

Sample size	No statistical methods were used to predetermine the number of participants or number of samples. Instead, this was guided by the clinical characteristics of the patient and the availability of samples at different time points during the course of treatment. The study consists of eight samples from six different time points of tumour progression in one patient.
Data exclusions	No data was excluded.
Replication	For two time points, two different biological samples were taken as effective replicates. Replicates confirmed presence of the same clones and were as such "successful".
Randomization	Not applicable, this study describes the evolution of lineage switch cancer in a single patient as a case report.
Blinding	Not applicable, this study describes the evolution of lineage switch cancer in a single patient as a case report.

Reporting for specific materials, systems and methods

We require information from authors about some types of materials, experimental systems and methods used in many studies. Here, indicate whether each material, system or method listed is relevant to your study. If you are not sure if a list item applies to your research, read the appropriate section before selecting a response.

Materials & experimental systems

n/a	Involved in the study
☒	☐ Antibodies
☒	☐ Eukaryotic cell lines
☒	☐ Palaeontology and archaeology
☒	☐ Animals and other organisms
☒	☐ Clinical data
☒	☐ Dual use research of concern

Methods

n/a	Involved in the study
☒	☐ ChIP-seq
☒	☐ Flow cytometry
☒	☐ MRI-based neuroimaging
